# Steady-state data-driven dynamic stability assessment in the Korean power system

**DOI:** 10.1038/s41598-025-90798-3

**Published:** 2025-03-05

**Authors:** Sungyoon Song, Sang-won Min, Seungmin Jung

**Affiliations:** 1 Tech University of Korea, 237, Sangidaehak-ro, Siheung-si, Gyeonggi-do South Korea; 2 Advanced Power Grid Research Department, Korea Electrotechnology Research Institute, Uiwang-si, Korea; 3 Department of Electrical and Computer Engineering, University of Seoul, Seoul, Korea

**Keywords:** Energy grids and networks, Electrical and electronic engineering

## Abstract

The extensive research on dynamic security assessment stability prediction has focused on data preprocessing techniques to improve accuracy because it was assumed that high-resolution postfault data exist. For practical users, the acquisition and application of high-resolution measurement data present significant challenges. Installing phasor measurement units on all power system nodes is deemed impractical due to high costs. In this work, we aimed to develop a rotor angle stability prediction model using steady-state data that can be easily generated from the current energy management system. Note that the steady-state measurement data refer to a pre-contingency operation condition characterized by real and reactive loads, generation levels, flows, as well as voltages and angles. The proposed framework comprises three stages: it finds physical meaning from the extended equal-area criterion to move away from the black-box approach, proposes a feature data extraction strategy to reduce the dimensionality of the input space in the support vector machine, and partition time-series power flow data by month to consider system topology changes. By utilizing 5-min-interval power flow data, unstable cases are determined, and two main feature data are extracted to train the support vector machine. The obtained results showed the effectiveness of the proposed framework in responding to a critical line fault event in real time.

## Introduction

The rapid increase in renewable energy resources around the world is a significant challenge in power system operations and planning^1^. Usually, the independent use of solar and wind power cannot meet the changing demands throughout the day. The acceleration of the duck curve phenomenon leads to various stability issues, including the shortage of reserve and system inertia^2^. Thus, an effective solution for managing an ecologically friendly renewable energy resource is highly required. Although various decentralized control and operation methods are currently under extensive research^3,4^, system operators must heavily rely on centralized control, such as the energy management system (EMS). Owing to the supervisory control and data acquisition (SCADA) capability, it continuously monitors and regulates the operational status of generators, substations, and transmission lines, making it an integral component of our energy infrastructure. Several researchers have incorporated centralized control ranging from a large-scale power grid to an isolated microgrid or home^5,6^. A general framework for the EMS is proposed in^7^, and the purely heuristic optimization-based EMS is reported in^8–10^. These systems ensure real-time optimization through dynamic programming^8^, Lagrangian relaxation^9^, and mixed-integer linear programming^10^. These studies mostly possess a central control room provided with the relevant information from the external system to determine the optimal dispatch of resources. In summary, the operation technology based on a centralized approach realizes minimum operational cost while satisfying technical constraints, offers a broad observability of the power grid, and finds global information using fully connected communications^11^. Moreover, the power grid has become a complex multienergy system, providing great opportunities for the centralized approach. Although the method can reduce flexibility and increase computational requirement, the current system introduces the parallel architecture-based computing unit^12^; thus, it is no longer vulnerable to a single point of failure.

**Fig. 1:**
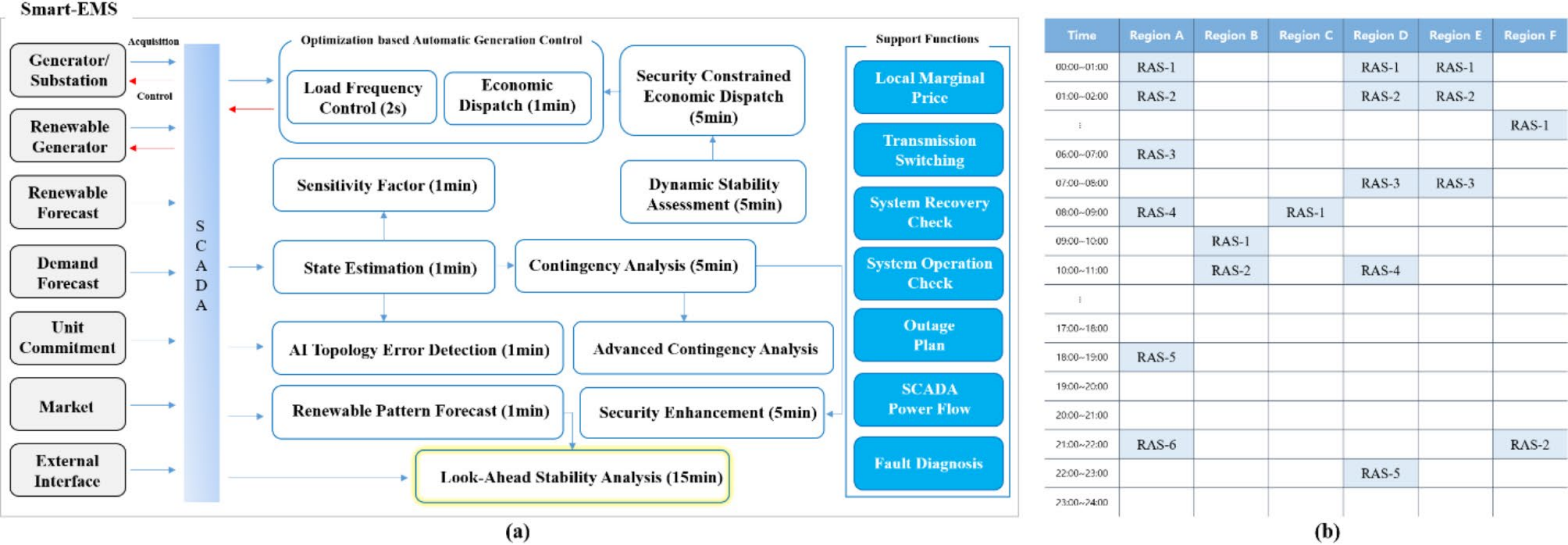
(**a**) Key functions of the Korean energy management system. (**b**) Remedial action schemes in South Korea.

The Korean EMS (K-EMS) implemented in the real power grid in October 2014 includes automatic generation control, load forecast, reserve margin assessment, optimal power flow, production cost assessment, and unit commitment. From 2023, South Korea is developing the Smart EMS, an enhanced version of K-EMS, melding cutting-edge technologies such as artificial intelligence and big data. The key functions of K-EMS are represented in Fig. 1a. One of the functions is look-ahead stability assessment (LSA), which aims to generate future network data six hours ahead by utilizing load forecast, renewable energy forecast, unit commitment, and circuit breaker outage planning data. The LNA module analyzes results up to 6 h in advance, concurrently generating results at 30-min intervals in the meantime. Dynamic security assessment (DSA), as well as static security assessment, is performed in the LSA.

## Literature review and motivation

In this section, we demonstrate the fundamental limitations of performing DSA in the LSA, with notable difficulty in generating dynamic simulation results in a given time. Regarding the LSA module, future stability assessment is vital for the secure operation of power grids. Security is a system attribute measured with respect to contingencies. The proposed LSA can address two different behaviors of a future power system, namely static and dynamic. Static security concerns the violation of operating variables at the steady-state post-contingency condition, whereas the DSA concerns the system stability during the transient period following a contingency. By using LSA results, several countermeasures have been proposed in real time to withstand future contingencies and take remedial action for security improvement. However, the main issue is the calculation time. The static and dynamic assessment calculations must be completed for 8 cases within 15 min. The DSA generally requires a significant computational time because of the numerical integration of a large system matrix, resulting in high offline computational costs^13^. Although remedial action schemes (RAS) are organized in accordance with the contingency list, performing DSA for every contingency list is impossible because of time constraints in current EMS. Accordingly, rule-based remedial action strategies for every contingency list are prepared^14^, as shown in Fig. 1b. Three inspiring solutions have been proposed to cope with these issues: (1) screening the contingency list^15^, (2) using the equivalent power system model^16^, and (3) utilizing machine learning to improve accuracy^17–21^. Although screening offers promising results using sensitivity, such as power transfer or line outage distribution factors, the sensitivity must be recalculated where rapid topology changes occur in the network. Thus, the first method heavily relies on the changing sensitivity matrix, which requires a massive measurement device. When unexpected fault events happen, this method cannot provide practical solutions as they are not designed to be immune to all possible events.

Further, equivalent power system models have been also heavily studied for DSA. The equivalent model is suitable for a small-scale power system because the speed governor and prime mover of the generating unit are generally simplified with the following assumptions: (1) system frequency is uniform, ignoring intermachine oscillations; (2) one equivalent generator inertia and one load damping factor are used. However, a maximum of 8% of the error occurs in the Spanish network with 19 generators compared to the full model-based dynamic simulation results^16^. As the size of the power grid increases, the error is bound to increase. Inaccurate results can initiate under frequency relay; thus, an accurate DSA is required in the real network. Accordingly, the equivalent model in the power system makes the verification of DSA results challenging.

**Fig. 2:**
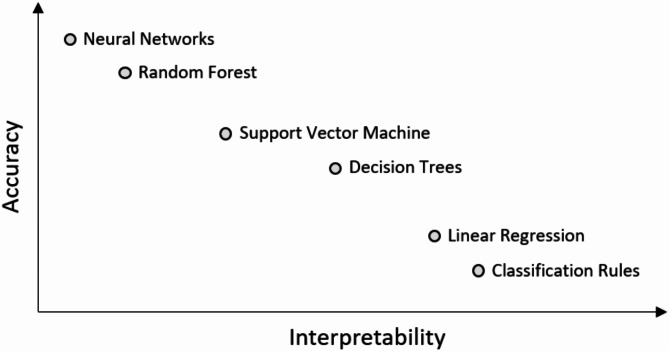
The trade-off between interpretability and accuracy of AI models^33^.

Last, learning-related studies have exhibited high potential in addressing the DSA problem from a data-driven perspective. However, two nontrivial issues emerge when they are applied to practical large-scale power systems. First, when the topology changes, one needs to retrain the neural network (NN) to learn a new solution mapping because large and complex NNs are often considered as black boxes, making it challenging to interpret their internal workings. Retraining the NN in real time^22^ or pretraining multiple NNs offline for all possible combinations of topology incurs significant data complexity. At least, the graph NN must be applied to produce a high-fidelity model; however, it also requires a minor relearning step because it changes edge features during topology processing. It is also computationally burdensome to independently train the model for each topology considering a high volume of model parameters to be tuned. Such a heavy computational burden can limit the applicability of the NN model in the EMS. Therefore, explainable AI model is required in industry area. So, we used a simple and rational model as a support vector machine (SVM) instead of the NN^23^. SVM has a certain level of interpretive ability, as shown in Fig. 2. The key point to note here is that trying to explain black-box models, rather than creating interpretable models from the start, is likely to perpetuate bad practices. While there is still ongoing debate, the^24^ highlights that efforts should prioritize building interpretable models from the outset. Furthermore, the notion that accuracy and interpretability exhibit a linear relationship, as shown below figure, is a subject of some debate.

We aimed to find physical meaning from several algebraic and differential equations to apply an effective training dataset. Highlighting the physical meaning of these attributes allows movement away from the black-box approach. Second, the existing studies related to the data-driven model often ignore the problem of how to access and acquire the quality data because they assume that all data are readily accessible. Thus, spectrum analysis and data preprocessing vary significantly^25,26^. Since the data being used vary, numerous preprocessing steps exist to deal with an exceptional case. Therefore, in this study, only power flow data without a preprocessing step were used for training the SVM model. We used the snapshot data from the EMS for practical use and feature data selection techniques considering that physically interpretable attributes were applied to overcome the curse of dimensionality of the input space. Notably, the snapshot data can be fully substituted with the scenario data, which refer to a pre-contingency operation condition characterized by real and reactive loads, generation levels, flows, as well as voltages and angles. The developers can easily manipulate the scenario data with their renewable profiles and unit commitment algorithms^27^. In other cases, their training data is based on the phasor measurement unit (PMUs) or the wide-area monitoring system. Although the prediction model using high-resolution data shows high accuracy, it moves from a low-dimensional to high-dimensional feature space. Installing PMUs on all nodes is also deemed impractical due to the prohibitively high associated costs. Furthermore, there are inherent limitations in preprocessing the large volume of high-resolution data. Therefore, the proposed framework can be easily integrated with general optimization models, such as optimal power flow, because the prediction model is trained by the steady-state measurement data. This advantage facilitates industrial adoption.

As mentioned earlier, the LSA aims to perform static and dynamic simulations for the upcoming six hours, executing evaluations such as flow and voltage violations, contingency analysis, fault current, reserve, inertia, frequency stability, rotor angle stability, and voltage stability. In particular, the rotor angle stability module is the main focus of this paper. In practical large-scale power grids, as numerous possible contingencies must be scanned, the rotor angle stability assessment increases the overall burden of computational cost. In contrast, frequency stability typically requires a single contingency, which is the event of the largest generator trip. Faced with the above-mentioned challenge, this work focuses on the rotor angle stability prediction model, which can help to reliably infer the stability decision boundary of a given system. Using machine learning techniques, we used the SVM for the real-time decision model. Many researchers already mentioned that the methods such as artificial NN, decision tree, and ensemble decision tree are difficult to improve^28^. Thus, our goal was not to outperform existing algorithms, as no new SVM algorithms have been proposed in this paper, but to considerably extend the set of feature datasets with physical meaning to improve the performance of existing machine learning tools. The core idea can be summarized as follows: (1) it proposes a monthly SVM model by partitioning the training power flow snapshot data, (2) it finds highly correlated feature data via physically interpretable attributes and ensures high accuracy regardless of the training model, and (3) it provides negligible online computation time in the EMS.

### Feature extraction scheme from power flow data

In this section, first, we introduce the feature extraction strategy to configure a generalized data mining procedure. In the proposed rotor angle stability prediction model, the extended equal-area criterion (EEAC) theory has been introduced to find physically interpretable attributes in power flow time-series data. In general, the power system can be cast in the form of a differential-algebraic (DAEs) discrete model with discrete events. For, example, Xue et al.^29^ describes a special case of the switching function described as

1 $$\left\{\begin{array}{l}\dot{x}=f\left(x,u,t\right)\\ 0=g\left(x,u,t\right)\end{array}.\right.$$

A switching occurs when $$g\left(x,u,t\right)=0$$. $$x$$ and $$u$$ are the dynamic state and input variables, respectively. $$f$$ and $$g$$ are the differential and algebraic equations, respectively. $$x$$ is continuously updated under the algebraic variable calculation at switching instants. Considering the initial conditions ($${x}_{0},{y}_{0})$$, Eq. (1) can be expressed by

2 $$\dot{x}=f\left[\left({x}_{0}+\Delta x\right),\left({u}_{0}+\Delta u\right)\right],$$

3 $$0=g\left[\left({x}_{0}+\Delta x\right),\left({u}_{0}+\Delta u\right)\right],$$

4 $$\Delta \dot{x}=\frac{\partial f}{\partial{x}_{1}}\Delta{x}_{1}+\cdots+\frac{\partial f}{\partial {x}_{n}}\Delta {x}_{n}+\frac{\partial f}{\partial {u}_{1}}\Delta {u}_{1}+\cdots +\frac{\partial f}{\partial {u}_{n}}\Delta {u}_{n},$$

5 $$\Delta y=\frac{\partial g}{\partial {x}_{1}}\Delta{x}_{1}+\cdots +\frac{\partial g}{\partial {x}_{n}}\Delta {x}_{n}+\frac{\partial g}{\partial {u}_{1}}\Delta {u}_{1}+\cdots +\frac{\partial g}{\partial{u}_{n}}\Delta{u}_{n}.$$

$\Delta y$ denotes the output variables, and the matrix size is determined by the number n. Equations (4) and (5) provide the stability of the oscillation modes of network results using the Lyapunov theory. In this work, we aimed to estimate $x$ using $y$. To reduce the input vector size, the EEAC, which coincides with the Lyapunov criterion, is analyzed in Section A. In our study, the DAEs were solved using PSS®E (Power System Simulator for Engineering) for the dynamic simulation of Korean power system.

(A) Extended equal-area criterion (EEAC)^29^

**Fig. 3:**
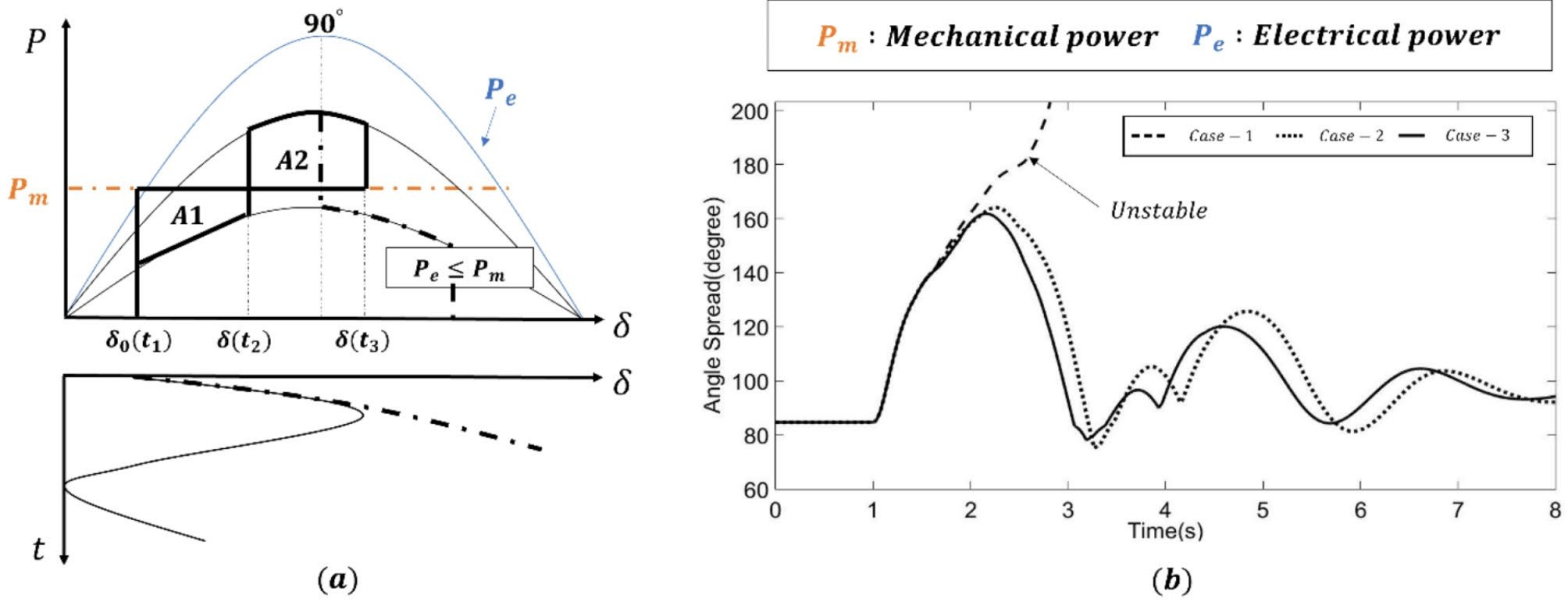
(**a**) Equal-area criterion analysis. (**b**) Unstable condition in rotor angle stability.

Multiple transient energy function-based rotor angle stability assessment methods were widely discussed for system security. By considering i-th multimachine power system dynamics, the center of angle (COA) is defined in (6). The COA can be illustrated as the inertia weighted average of generator rotor angles:

6 $${\delta}_{ce}=\frac{1}{{H}_{e}}\sum_{i=1}^{n}{H}_{i}{\delta}_{i},$$

where ${H}_{e}$ is the total inertia. The rotor angle can be calculated by subtracting the critical generator’s rotor angle and the remaining generator’s rotor angle. The critical generators cause loss of synchronism. Then, the EEAC concept to the one machine infinite bus (OMIB) equivalent can be represented by

7 $${\delta=\delta}_{i}-{\delta}_{s}, \quad \frac{H}{{w}_{0}}\frac{{d}^{2}\delta}{{dt}^{2}}={P}_{m}-{P}_{e},$$

where $\delta$ and $H$ stand for the rotor angle and inertia related to the equivalent OMIB system. $H$ is calculated by ${(H}_{i}{H}_{s})/{H}_{e}$, and ${w}_{0}$ is the synchronous speed. ${P}_{m}$ and ${P}_{e}$ are the mechanical and electrical power of the equivalent OMIB, respectively. EEAC can be represented by multiplying $2d\delta/dt$ by the swing equation as follows:

8 $$\frac{H}{{w_{0} }}\left( {\frac{{d\delta }}{{dt}}} \right)^{2} = 2\int\limits_{{\delta _{0} }}^{\delta } {(P_{m} - P_{e} )d\delta ,}$$

9 $$\frac{d\delta}{dt}\propto \pm \sqrt{\int{P}_{m}-{P}_{e}d\delta},$$

where ${\delta}_{0}$ is the pre-contingency rotor angle in the equivalent generator. In summary, the rotor angle variation is linked to the area between ${A}_{1}$ and ${A}_{2}$. For example, $\delta$ increases with increasing ${P}_{m}$ or by reducing ${P}_{e}$, as shown in (9). The variations in the system rotor angle $\delta$ are coupled with the area between ${P}_{m}$ and ${P}_{e}$ in the $\delta-P$ plane, as shown in Fig. 3a.

The accelerating area is defined as ${A}_{1}$ when ${P}_{m}>{P}_{e}$, as shown in Fig. 3a. Meanwhile, the decelerating area, ${A}_{2}$, occurs when ${P}_{m}<{P}_{e}$. In other words, ${A}_{1}$ is the amount of energy of generators that increases during the fault, and ${A}_{2}$ is the maximum energy that the power system can dissipate in the postfault condition. ${A}_{1}$ and ${A}_{2}$ can be expressed by

10 $$A_{1} = \int\limits_{{\delta _{0} \left( {t_{1} } \right)}}^{{\delta \left( {t_{2} } \right)}} {\left( {P_{m} - P_{{ea}} \left( \delta \right)} \right)d\delta ,}$$

11 $$A_{2} = \int\limits_{{\delta \left( {t_{2} } \right)}}^{{\delta \left( {t_{3} } \right)}} {\left( {P_{{eb}} \left( \delta \right) - P_{m} } \right)d\delta ,}$$

where $ea$ and $eb$ are the electrical power during and after the clearance of faults, and ${t}_{1}$ and ${t}_{2}$ represent the fault time and the fault clearance time, respectively. Last, ${t}_{3}$ is the time when the system reaches the unstable equilibrium point. The system is unstable when the sum of ${A}_{1}$ and ${A}_{2}$ becomes negative, and the rotor angle starts to increase, as shown in the first case in Fig. 3b. By introducing fast fault clearance, the system is stable when ${A}_{1}={A}_{2}$, and the rotor angle starts to decrease. Further expansion of (12) can be achieved by (8) as follows:

12 $$\frac{H}{{w_{0} }}\left( {\frac{{d\delta }}{{dt}}} \right)^{2} = 2\int\limits_{{\delta _{0} }}^{\delta } {\left( {P_{m} - \frac{{EVsin\left( \delta \right)}}{{X_{T} + X_{e} }}} \right)} d\delta .$$

Here, E is the internal voltage of the generator, and ${X}_{T}$ and ${X}_{e}$ are respectively the reactance of the equivalent transformer reactance and the reactance of transmission lines. Considering the term of E, fast reactive power injection is helpful to improve stability.

**Table 1:** Korean power system information.

	2025 (Off-Peak)	2025 (Peak)	2026 (Off-Peak)	2026 (Peak)
Reserve (MW)	1995.58	3116.54	2484.58	3378.56
Turn-on generator	58	166	64	166
Average rated capacity of generators (MVA)	726.48	518.56	743.59	512.54
Power flow to load center (GW)	10.3	24.15	6.38	17.56

**Fig. 4:**
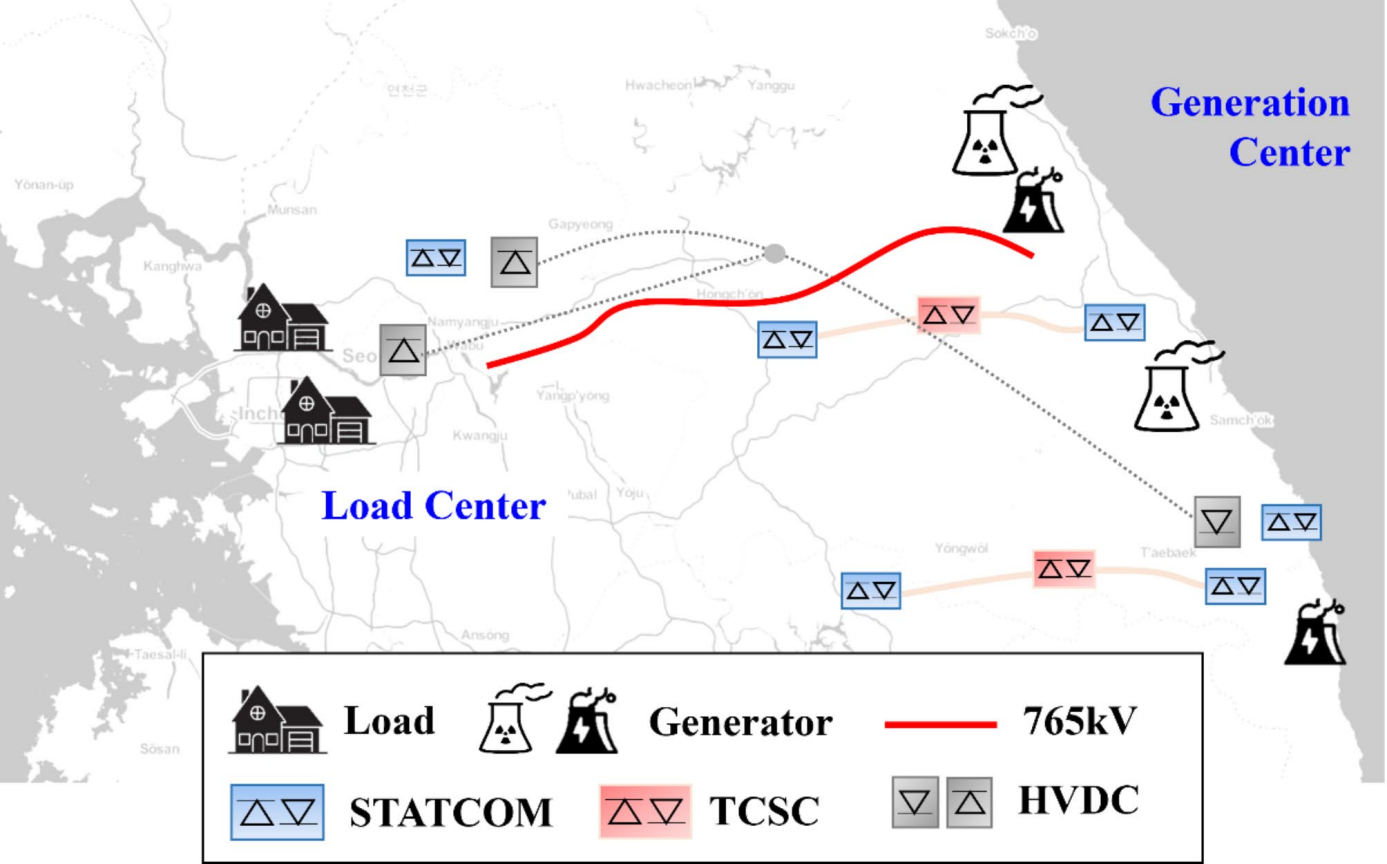
Korean power system configuration.

The basic information about the Korean power system is summarized in the Table 1. When demand is low, it is approximately 60 GW, while during peak demand on weekdays, it reaches around 100 GW. An extremely large load center exists in Korean power systems in the metropolitan area and generation center in the seacoast as illustrated in Fig. 4. Figure 4 was generated using Python, an open-source programming language, to visualize the map of South Korea. The visualization was created with the Python module *Folium*, which is based on leaflet.js. *Folium* enables the creation of maps by processing latitude and longitude data in JSON format for direct visualization. The direction of massive power flow is always from the generation center to the load center. The trip of 765 kV ac transmission lines between two areas generally accelerates the generator angle. Projections suggest that 6.4 GW of generation constraints may occur this year alone. The reason for introducing trip of 765 kV lines is that, according to Korea’s reliability standards, all transmission lines must meet the N-2 contingency criteria. While countries like India or United States apply varying reliability standards based on the importance of the transmission lines, Korea requires N-2 contingency analysis for all lines. In the case of trip of 765 kV lines, it frequently results in generator angle instability. To prevent rotor angle instability, the government tries to install a HVDC (high-voltage direct current) or additional ac transmission line on the power grid, but public acceptance is also a growing concern nowadays.

With the peak load condition, the power flow at each ac transmission line approaches its nominal rating, and the maximum power transfer between two areas forces the DSA of all possible events in real time. The difficulty of solving this problem motivated the development of an online rotor angle prediction model. The strategy used to pick a stability decision boundary must be learned whenever new grid operating conditions are considered.

(B) Feature extraction scheme with power flow data

From the time-series power flow data, we can generate 300 + attributes per one snapshot data, such as voltage, angle, power, generator reactance, and generator inertia. Notably, we try to find physical meaning from the EEAC. Based on Eqs. (10)–(12), the initial generator angle ${{\updelta}}_{0}\left({t}_{1}\right)$ is the most important index because large ${{\updelta}}_{0}$ can easily lead to the acceleration area, which is over 90 degrees. If ${P}_{e}$ is larger than ${P}_{m}$ and the generator angle is larger than 90 degrees, the loss of synchronism arises. In other words, ${{\updelta}}_{0}$ provides key insights into how to reduce the size of the input vector. ${{\updelta}}_{0}$ is the pre-contingency rotor angle in the equivalent generator, and it determines rotor speed and initial active power generation in the generation center. It is also linked to the power flow sum between sending and receiving areas. Ten types of possible candidate feature data related to ${{\updelta}}_{0}$ were determined, as shown in Table 2. To reduce the size of the input vector, feature data extraction strategy was performed by the accuracy test in this Section. Considering two final data, namely ${F}_{n}$ and ${F}_{p}$, we select $\{2{(F}_{n},{F}_{p})\times 12\times 30\times 288\}$ out of $\{{10(F}_{1},\cdots,{F}_{10})\times12\times30\times288$ because the data comprised of 5-min-interval power flow data for one year.

**Table 2:** Feature candidate dataset (GC: Generation Center, LC: load Center).

Feature	Feature description (before ${t}_{1}$)
${F}_{1}$	Tie-line flow between GC and LC
${F}_{2}$	Total generation at GC
${F}_{3}$	Total generation at LC
${F}_{4}$	Number of turn-on generators at GC
${F}_{5}$	Number of turn-on generators at LC
${F}_{6}$	Load active power and reactive power demand in LC
${F}_{7}$	Load active power and reactive power demand in GC
${F}_{8}$	Bus angle difference between GC and LC
${F}_{9}$	STATCOM reactive power margin at GC
${F}_{10}$	Number of turn-on STATCOM at GC

**Fig. 5:**
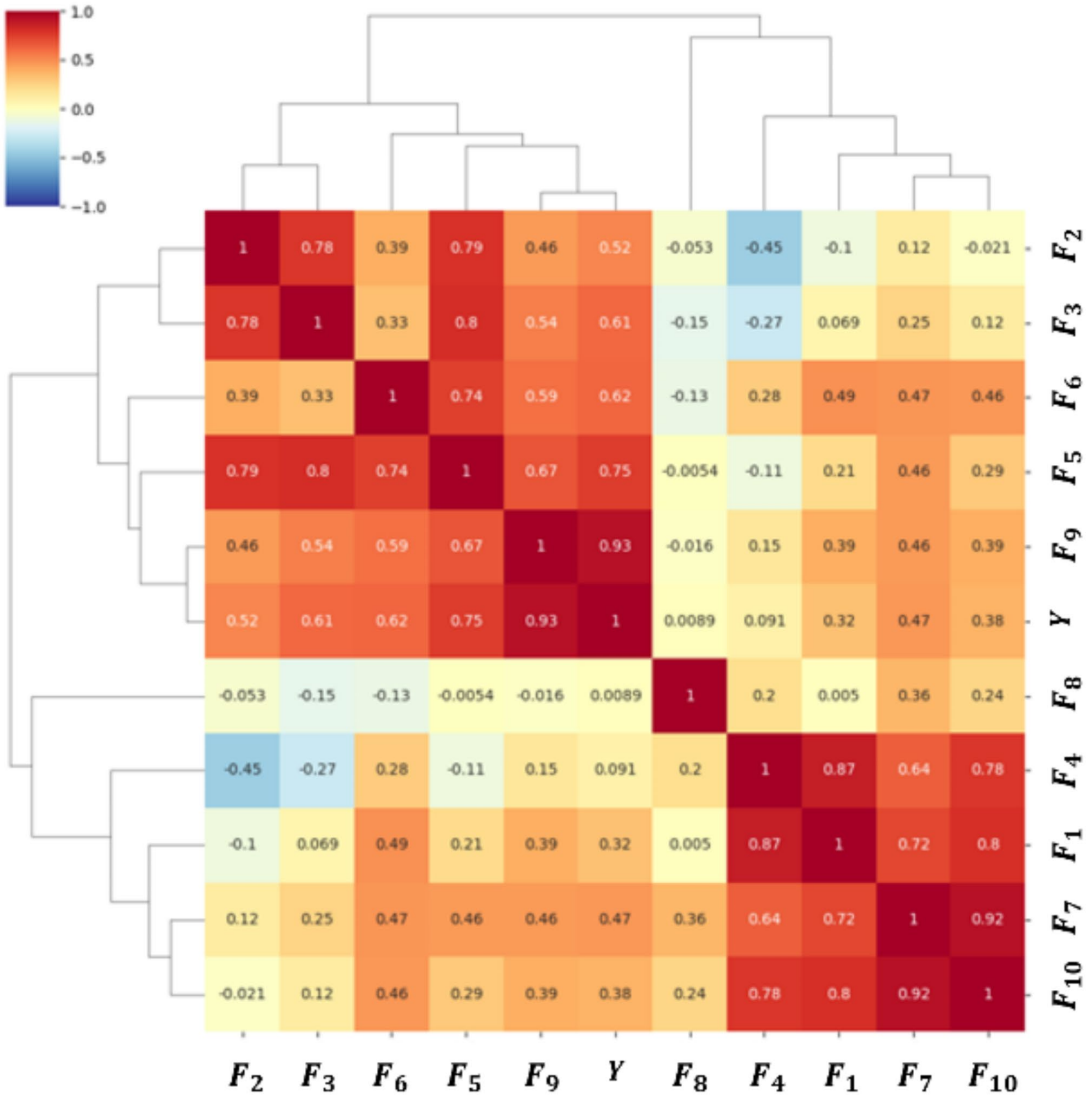
Sensitivity analysis for physically explainable attributes selection.

Traditionally, clustering techniques are used to reduce the number of observations from correlations. However, applying these approaches to all power flow data leads to spurious correlations and inappropriate clustering. We focus on the input dimension number, so a more physically meaningful state-space reduction approach is necessary. In order to train the SVM model with two selected data points out of a total of ten, a sensitivity analysis on the Y-values was conducted as shown in Fig. 5. The heatmap guided the selection of the top two X variables (${F}_{n}, {F}_{p}$) exhibiting the highest sensitivity.

**Fig. 6:**
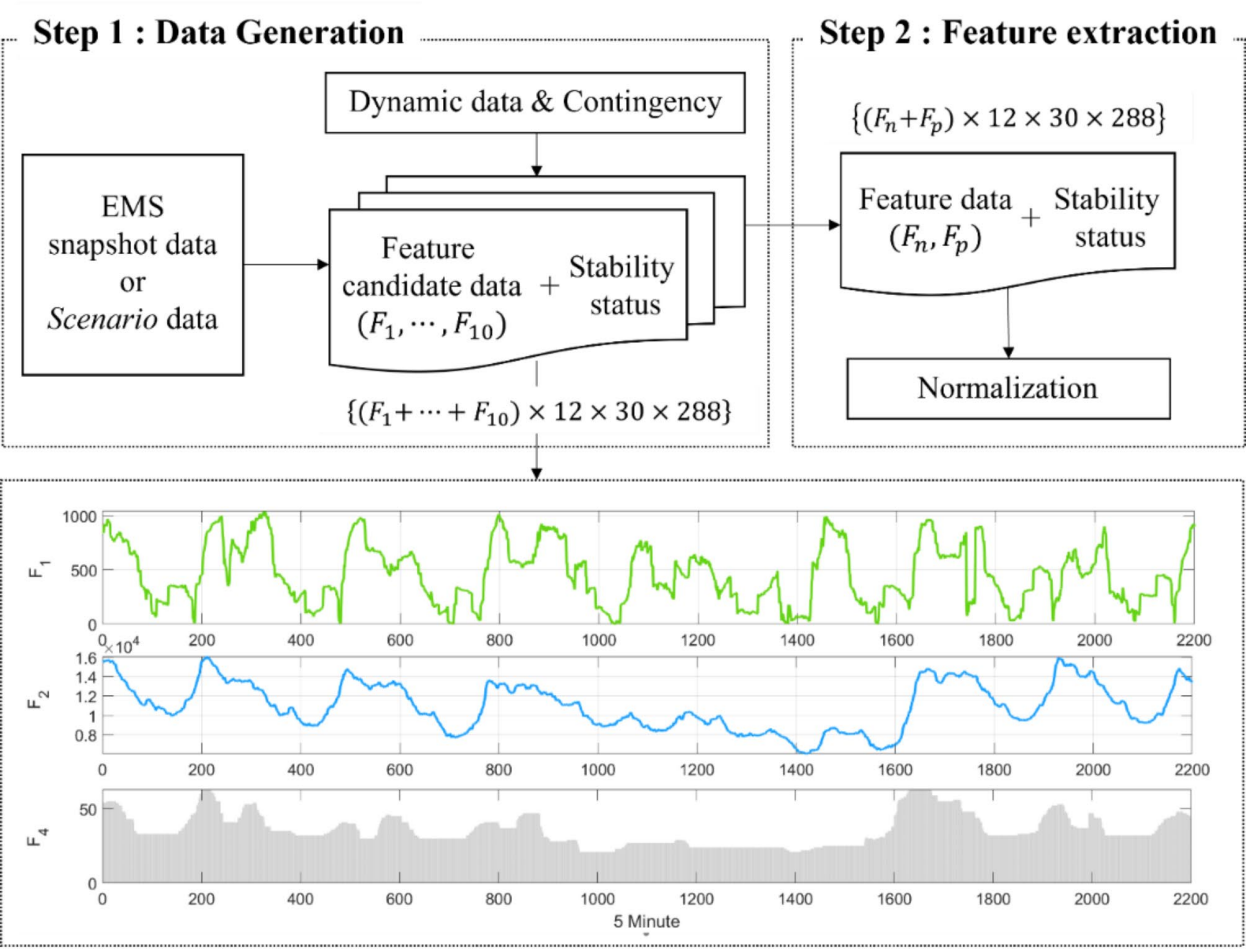
Feature dataset extraction strategy.

And, we split observations into monthly groups corresponding to real historical snapshot data to consider topology changes^30,31^ and find optimal combinations among the candidate feature dataset, as illustrated in Fig. 6. Applying this approach to reduced observation vectors ensures that a small number of parameters and correlations does not impede the learning process.

State-of-the-art research methods such as Kullback-Leibler divergence can capture the dynamic trajectory, such as voltage, angle, and frequency^32^. At the same time, Daubechies and Symlet transforms were suggested in recent prediction model for high-resolution data^25^. By using trajectory data, it is possible to derive a single SVM-based stability predictor and generalize the stability features to all types of network dynamics and novel operating conditions. However, the objective of our strategy was to avoid using a dynamic simulation dataset. Therefore, trajectory feature extraction was not performed in this study.

## Training data with a support vector machine

In this paper, we utilized the widely used SVM, which allows the linear or nonlinear function assumption in a high-dimensional characteristic space. In simple terms, SVM works by mapping input data into a higher-dimensional feature space where it becomes easier to separate classes with a clear boundary. Instead of finding a separating line or curve in the original input space, SVM identifies an optimal hyperplane in this transformed space. This hyperplane maximizes the margin between the classes, ensuring better generalization. By using kernel functions, SVM can handle complex relationships within the data, making it a powerful tool for both linear and nonlinear problems. Because the SVM is one of the most powerful classifiers in terms of the trade-off between effectiveness and complexity, it was selected to implement the proposed strategy in the Python code.

The primary reason for using the SVM model is its ability to identify and explain the most significant physical parameters through sensitivity analysis, unlike neural network models that act as black boxes by learning from all available input data. Additionally, SVM can perform nonlinear classification using only two or three selected key parameters. Limiting time-series data inputs to two or three ensures that EMS operations can function in real-time. High-sampling-rate data, even with just two inputs, can result in excessive transmission delays to the EMS. For these reasons, we selected SVM as the predictive model. The algorithm requires a database that well represent the class value so that it can effectively classify new instances and not overlook the less representative classes. So, for a two-class problem, a good representation of operating conditions on both sides of the class boundary is required. The database must contain operating conditions closer to the security boundary, enabling clear-cut decision-making regarding the acceptability of any operating condition. Therefore, we need monthly training data that reflect system topology changes. A complete survey of the SVM is beyond the scope of this study^28^; we have provided a short summary here. Suppose that there exists a hyperplane, and the constraints that the hyperplane must separate the points,

13 $${x}_{i}\cdot \mathbf{w}+b=0,$$

14 $${y}_{i}\left({x}_{i}\cdot\mathbf{w}+b\right)\ge0,$$

with parameters ($\mathbf{w}$, $b$), separating positive form negative examples. The optimization problem is to minimize $\left\| {\text{w}} \right\|$ under the distance constraint, and the goal of the training is to find the hyperplane that best separates the training data. The proposed filter training data is represented by

15 $$S=\left\{\left({F}_{ij},{Y}_{ij}\right)\left|{F}_{ij}\right.=\left\{{F}_{1j},\dots ,{F}_{10j},\right\}\right\}, \quad for\;\;1\le i\le n.$$

Here, ${X}_{i}$ is the input of case $i$, i.e., the 5-min-interval power flow snapshot data of the $i$-$th$ case, including ${F}_{1j}$–${F}_{10j}$ in Table 2. $j\in\{1,2\dots 11, 12\}$ means respective month to split training data, and ${Y}_{ij}\in\{-\text{1,1}\}$ denotes the rotor angle stability output of case i in the j-th month. The class label ${Y}_{ij}$ comprises $-1 \to \text{unstable},+1 \to \text{stable}$. ${Y}_{ij}$ can be obtained from DSA using PSS®E, and it is determined by a specific stability criterion. A widely used transient stability criterion based on rotor swings is implemented to classify the results, as follows:

16 $$\eta=\frac{{360}^{\circ}-{\left|\Delta \delta\right|}_{max}}{{360}^{\circ}+{\left|\Delta\delta \right|}_{max}}> 0 \Rightarrow \text{stable}.$$

**Fig. 7:**
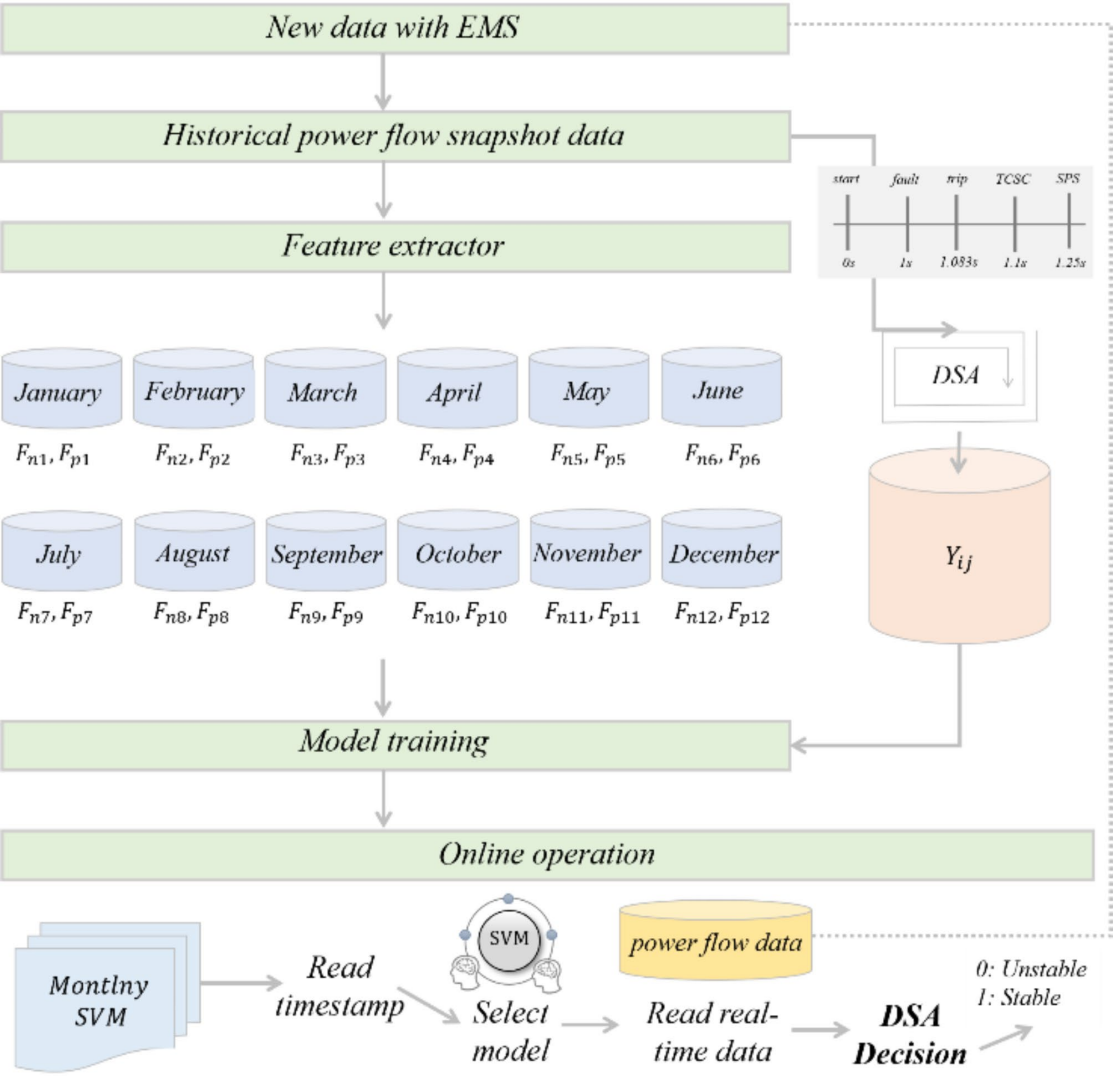
Flow chart of the power flow data-based rotor angle prediction model.

Here, $\eta$ denotes the transient stability index, and ${\left|\Delta\delta\right|}_{max}$ is the maximum absolute rotor angle difference between any two generators during the transient process. In dynamic simulations, the angle spread, which is the difference between the largest and smallest machine angles, is used since there are over 400 generators in South Korea. Unlike the power flow data (denoted as ${X}_{ij}$), which can be quickly acquired from the EMS, obtaining the DSA results as ${Y}_{ij}$ incurs a computational cost. To attach the ${Y}_{ij}$ label, we conducted 207,360 dynamic simulations, which required at least three months of data organization. The contingency timelines were as follows: (1) simulation start: 0 s, (2) apply 3-phase fault at 765 kV bus: 1 s, (3) remove fault and trip lines: 1.0833s, (4) thyristor-controlled series compensator (TCSC) action: 1.1 s, (5) special protection system (generator trip) activate at 1.25 s, and (6) simulation end: 10 s. If an instability condition can be driven by the proposed model sufficiently early, preventive actions such as generation power cut, reactive power injection, or transmission line switching can be taken. The flow chart for training and application of the proposed model is illustrated in Fig. 7.

## Results and discussion

**Fig. 8:**
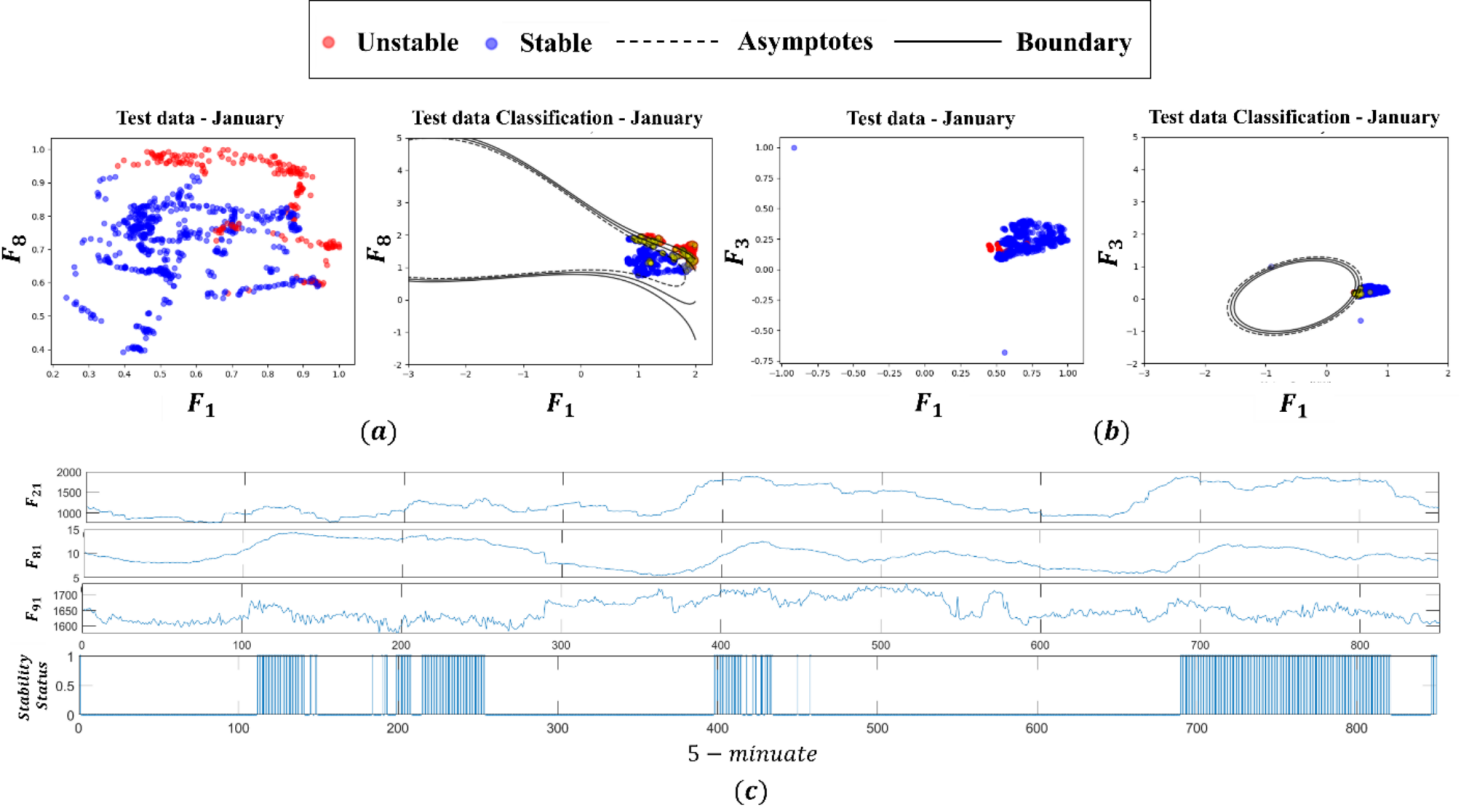
Training results: (**a**) Input pairs of ${F}_{1}$ and ${F}_{8}$ with January data, (**b**) Input pairs of ${F}_{1}$ and ${F}_{3}$, (**c**) Feature dataset.

To evaluate the performance of the overall framework on the data-driven rotor angle prediction model, the accuracy metric was evaluated. The accuracy metric (%) mentioned here refers to the evaluation of whether the rotor angle stability results obtained using a prediction model match the outcomes derived from real EMS engine. A comparison test was designed to demonstrate the proposed framework. The Korean power system data were examined for practical testing in real large-scale systems. The datasets are obtained by EMS, and all computations as well as data processing were conducted on a PC configured with an Intel Core i7 CPU and 24 GB RAM. The hyperparameters were determined as $C$ = 100 and gamma = 0.1. The total number of samples was 1,500–2,500 in each month (the ratio of stable to unstable is approximately 8:1), 80% of the samples were the training data, and 20% of them were the testing data, as shown in Fig. 8. And, the test data was obtained by using the last 20% of the data from each month’s dataset. The training and test data were applied using one year of data at 5-minute intervals. The real load and generation profiles for one year enhance the richness of training databases. The reason for the varying number of monthly sample data is that we excluded both cases in which the dynamic simulation did not converge and power flow data were missing. In this paper, we have defined this data as measurement error data. After the training, the accuracy of the proposed monthly SVM model is illustrated in Table 3. In addition to accuracy, two additional metrics, Precision and Recall, were analyzed to better evaluate the performance of the model. Precision represents how many of the predicted positive cases were actually correct, focusing on the model’s ability to reduce false positives. Recall, on the other hand, shows how many of the actual positive cases were correctly identified, emphasizing the model’s ability to detect positive cases. These metrics were included to address the limitations of accuracy, especially when dealing with imbalanced datasets, providing a clearer and more balanced understanding of the model’s performance.

The system dynamics were studied using PSS®E by simulating 765-kV line tripping and a three-phase ground fault. In other words, the major contingency scenario was applied to the real power grid to find the stability boundary. The model was designed to enable system operators to evaluate the stability boundary from a more conservative perspective. For each simulation, a stability status (0 or 1) was recorded by (16). In fact, contingency did not incorporate remedial control actions, such as the static synchronous compensator control, which can improve rotor angle stability or the TCSC action. From June to August, a high volume of converged state estimation data and very few measurement error data can be observed, as shown in Table 3. In November and December, a notable decrease in model accuracy was observed because the power flow data was not converged due to wrong data acquisition from SCADA. This issue leads to non-converged DSA results, further impacting model accuracy.

**Table 3:** Test data configuration and model accuracy.

Month	Number of measurement error cases	Prediction accuracy (%)	Precision	Recall
1	138	91.681	0.889	0.862
2	232	93.152	0.885	0.839
3	205	92.224	0.881	0.84
4	52	96.584	0.955	0.945
5	10	94.512	0.943	0.941
6	7	95.812	0.957	0.955
7	8	96.541	0.964	0.962
8	4	96.512	0.964	0.964
9	63	97.261	0.96	0.947
10	257	91.258	0.861	0.81
11	419	88.987	0.806	0.722
12	573	87.515	0.8	0.7

The original DSA time for screening one contingency was calculated to be 3.9 s based on the full Korean power system model. It can be observed that the time cost significantly increases to screen every contingency in LSA. After obtaining the feature dataset, the training and simulation times for one contingency were calculated as 7 s and 4.3${e}^{-3}$s. The training and decision-making time are significantly reduced. Notably, our method, which solely relies on static data, exhibits reduced accuracy compared to earlier methods, as shown in Table 2 (average model accuracy is 93.5%). Nevertheless, the proposed model demonstrates its capability as a well-made prediction model because the only steady-state measurement data was used. Furthermore, well-converged power flow data can also increase the model accuracy.

**Table 4:** Accuracy comparison for different candidate combinations of the feature dataset using January data.

Feature dataset	Prediction accuracy (%)	Precision	Recall
{${F}_{11}\&{F}_{21}$}	82.0991	0.771	0.721
{${F}_{11}\&{F}_{31}$}	84.5694	0.796	0.746
$\{{F}_{11}\&{F}_{61}$}	77.5186	0.725	0.675
${\{F}_{11}\&{F}_{91}$}	76.4215	0.714	0.664
$\{{F}_{11}\&{F}_{81}$}	91.6815	0.867	0.817
$\{{F}_{21}\&{F}_{41}$}	79.7821	0.748	0.698
$\{{F}_{21}\&{F}_{91}$}	82.46	0.775	0.725
$\{{F}_{41}\&{F}_{51}$}	74.8631	0.7	0.649
$\{{F}_{81}\&{F}_{91}$}	76.4215	0.714	0.664

We arbitrarily paired two feature datasets that exhibited physical relevance, and the selected results are shown in Table 4. Two datasets were paired and tested with the stability status. The combination of the tie-line flow ($${F}_{1}$$) and bus angle difference ($${F}_{8})$$ outperforms the other feature data with an accuracy of 91.6815%. The two types of data are correlated with the pre-contingency rotor angle $${\delta}_{0}$$ as it determines the total amount of transferred active power between two areas. Contrary to initial expectations, the reactive power margin in STATCOM did not substantially contribute to the accuracy. The results may arise from the failure to include the trajectory data of the reactive power output after a contingency. As mentioned earlier, the magnitude variations of dynamic states can be captured via various trajectory feature extraction methods. However, our method relies only on the steady-state measurement data, posing a fundamental limitation to the usage of dynamic data sets. Notably, the combination of the tie-line flow $$\left({F}_{1}\right)$$ and generation amount at the load center ($${F}_{3}$$) fails to maintain its accuracy in a specific month; however, it performs better in some cases, having a relatively high score. This implies that $${F}_{1}$$, $${F}_{3}$$, and $${F}_{8}$$ control the contribution of the clear-cut decision boundary. Moreover, the accuracy proves that the application of the proposed learning method can be utilized in the real power grid. When performing the dynamic simulation using snapshot data, the network does not converge in some cases. In other words, useless DSA results indicating discrepancies are mixed in the training dataset, leading to inevitable minor errors. Accordingly, the trained model can substitute complex rule-based remedial action strategies. Additionally, the model performs well every month.

**Fig. 9:**
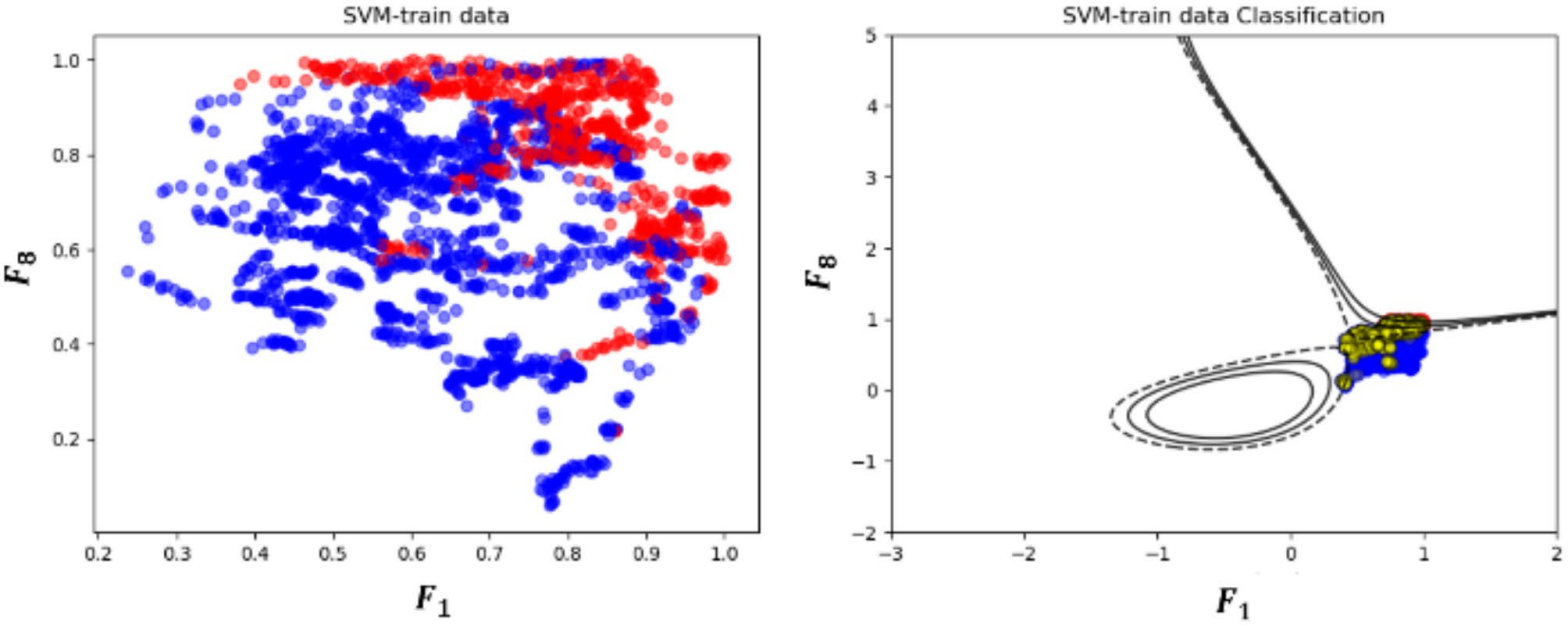
Training results with three-month merging data.

**Table 5:** Accuracy results of monthly data versus combined monthly data.

Feature dataset	Prediction accuracy (%)
{${F}_{11}\&{F}_{81}\}$	91.6815
{${F}_{11}\&{F}_{81}\}+{\{F}_{12}\&{X}_{82}\}$	88.8515
$\left\{{F}_{11}\&{F}_{81}\right\}+\left\{{F}_{12}\&{F}_{82}\right\}+\{{F}_{13}\&{F}_{84}\}$	84.6941

**Fig. 10:**
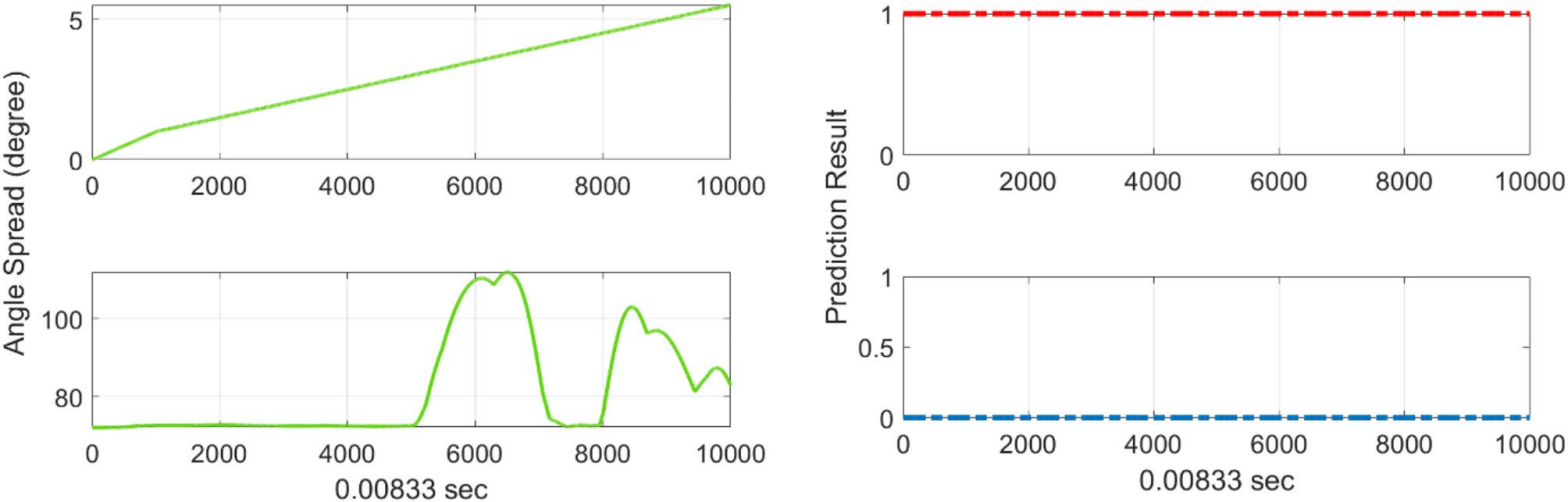
Unit test results (**a**) angle spread (**b**) rotor angle stability results with SVM model.

Furthermore, the results of combining multiple monthly data show low performance, as shown in Fig. 9; Table 5. Since the system operation varies greatly every month because of the demand pattern and the outage scheduling of generators, the SVM model must also be trained on monthly data to increase its accuracy. The comparison of the accuracy metrics of nonmonthly and monthly data showed that the latter model performs better in the decision boundary. The nonmonthly data exhibit complex nonlinear boundary lines, as represented by the black line in Fig. 9. The 3-month merged data showed that the accuracy decreased by 7% in SVM model. This result indicates that the power system operation plan is determined by seasonal characteristics, such as a synchronous generator outage plan and renewable patterns. By utilizing monthly training data, the proposed prediction model inherently considers the topology change. Instead of using a high-resolution database, we opted for power flow data and highlighted physical attributes in the EEAC. It leads to more general and simpler predictor, which retains the physical meaning. Considering the ten physically explainable attributes that describe the EEAC, we agree that this approach can be expanded to other dynamic predictor such as frequency and system inertia. In summary, for unseen scenarios i.e., renewable change and topology change scenarios, the proposed prediction model can give precise prediction results, as shown in Fig. 10. Note that, the timestep was selected as 0.0083 for the numerical integration in the dynamic simulation.

### Conclusions and future work

Existing methods for feature data extraction require significant expertise for signal processing because high-resolution measurement or dynamic simulation data are generally implemented. In this paper, therefore, we only used power flow data with the physical interpretation of the EEAC. The proposed strategy can be adopted in the industry because phasor unit measurement data are not required, offering an alternative ready-to-use power system operations. To extract the feature data, ten types of candidate data were simulated during the three-phase fault and line tripping scenario. The most correlated feature dates were determined according to their accuracy percentages and then classified into two categories using the SVM. The proposed model was built using Python scripts and automatically scrapes the EMS database. The two main findings can be stated as follows: (1) steady-state measures as power flow result in similar performance when fed with high-resolution data; (2) monthly data can reduce the dimensionality of the input space and increase accuracy compared to one-year data. A single SVM-based stability predictor can be derived using the trajectory data; however, static data must be partitioned by month to consider seasonal topology changes. In future works, we will combine the optimal power flow module, which has been widely studied to address the economic side. The transient stability of the postfault system was determined by the proposed model, and the power flow solution corresponded to the prefault operating point. Thus, the DAE-constrained optimization problem can be solved directly. Further, dimensionality reduction techniques such as PCA (Principal Component Analysis) and t-SNE (t-distributed Stochastic Neighbor Embedding) are being explored in ongoing research; however, these methods do not result in explainable AI models. Under the present circumstances, a more practical approach is to identify the most influential parameters through sensitivity analysis based on physical differential equations and to make predictions using only 2–3 key input variables.

## Data Availability

The datasets generated and/or analysed during the current study are not publicly available due to confidentiality regulations under the Korean Energy Management System but are available from the corresponding author on reasonable request.
